# Delayed gadolinium-enhanced MRI of the fibrocartilage disc of the temporomandibular joint – a feasibility study

**DOI:** 10.1016/j.mri.2014.08.008

**Published:** 2014-12

**Authors:** Elisabeth Pittschieler, Pavol Szomolanyi, Martina Schmid-Schwap, Michael Weber, Monika Egerbacher, Hannes Traxler, Siegfried Trattnig

**Affiliations:** a Department of Orthodontics, Bernhard Gottlieb University Clinic of Dentistry, Medical University of Vienna, Sensengasse 2a, 1090 Vienna, Austria; b High Field MR Centre, Department of Biomedical Imaging and Image-guided Therapy, Medical University of Vienna, Waehringer Guertel 18-20, 1090 Vienna, Austria; c Department of Imaging Methods, Institute of Measurement Science, Slovak Academy of Sciences, Dubravska cesta 9, 84219 Bratislava, Slovakia; d Department of Prosthodontics, Bernhard Gottlieb University Clinic of Dentistry, Medical University of Vienna, Sensengasse 2a, 1090 Vienna, Austria; e Department of Biomedical Imaging and Image-guided Therapy, Medical University of Vienna, Waehringer Guertel 18-20, 1090 Vienna, Austria; f Institute for Anatomy, Histology and Embryology, University of Veterinary Medicine, Veterinärplatz 1, 1210 Vienna, Austria; g Center for Anatomy and Cell Biology, Department of Systematic Anatomy, Medical University of Vienna, Währingerstrasse 13, 1090 Vienna, Austria; h Austrian Cluster for Tissue Regeneration, Ludwig Boltzmann Institute for Clinical and Experimental Traumatology, Donaueschingenstraße 13, 1200 Vienna, Austria

**Keywords:** dGEMRIC, Temporomandibular joint, Fibrocartilage disc, MRI at 3 T, Glycosaminoglycan in temporomandibular joint

## Abstract

**Objective:**

To 1) test the feasibility of delayed Gadolinium-Enhanced Magnetic Resonance Imaging of Cartilage (dGEMRIC) at 3 T in the temporomandibular joint (TMJ) and 2) to determine the optimal delay for measurements of the TMJ disc after i.v. contrast agent (CA) administration.

**Design:**

MRI of the right and left TMJ of six asymptomatic volunteers was performed at 3 T using a dedicated coil. 2D inversion recovery (2D-IR) sequences were performed at 4 time points covering 120 minutes and 3D gradient-echo (3D GRE) dual flip-angle sequences were performed at 14 time points covering 130 minutes after the administration of 0.2 mmol/kg of Gd-diethylenetriamine pentaacetic acid ion (Gd-DTPA)^2-^, i.e., 0.4 mL of Magnevist™ per kg body weight. Pair-wise tests were used to assess differences between pre-and post-contrast T1 values.

**Results:**

2D-IR sequences showed a statistically significant drop (*p* < 0.001) in T1 values after i.v. CA administration. The T1 drop of 50% was reached 60 minutes after bolus injection in the TMJ disc. The 3D GRE dual flip-angle sequences confirmed these results and show plateau of T1 after 60 minutes.

**Conclusions:**

T1(Gd) maps calculated from dGEMRIC data allow *in vivo* assessment of the fibrocartilage disc of the TMJ. The recommended measurement time for dGEMRIC in the TMJ after i.v. CA administration is from 60 to 120 minutes.

## 1 Introduction

The fibrocartilaginous disc of the temporomandibular joint (TMJ) is suspended between the superior (glenoid fossa, os temporale) and inferior (mandibular condyle, mandibula) articulating surfaces of the TMJ and has several important functions, one of which is the dissipation and distribution of masticatory loads [1], [2]. Eighty to ninety percent of the dry weight of the TMJ disc is collagen [3], and about 1% of the dry weight consists of glycosaminoglycans (GAGs) [4]. The TMJ disc region shows more highly sulfated GAG and collagen content than the attachments of the disc [5].

Temporomandibular disorders (TMDs) are widespread, occurring in 8% to 44% of individuals examined in epidemiologic and clinical studies, thus affecting millions of people [6], [7], [8]. The prevalence of clinical symptoms of TMD in an American population was about 6 - 12% [9]. However, there is a peak occurrence between 20 and 40 years of age [10]. One part of TMD is the articular disorders (internal derangement) which is a noninflammatory arthropathy and equates changes in the disc-condyle relationship [11], [12]. A recent study among 6-8 year old children showed that 35% of these children had at least one clinical sign of TMD. [13] The TMJ also plays a role in posture and body biostatics [14].

T1 mapping of cartilage after delayed gadolinium diethylenetriaminepentaacetate acid ion (Gd-DTPA)^2-^ enhancement, called delayed Gadolinium-Enhanced Magnetic Resonance Imaging of Cartilage (dGEMRIC), has emerged as a promising biochemical Magnetic Resonance Imaging (MRI) technique for the quantitative evaluation of articular cartilage [15]. The dGEMRIC has been validated as a clinically useful tool for the relative glycosaminoglycan content of repair issue after various types of chondrocyte transplantation [16]. Furthermore, in combination with T2 mapping a dGEMRIC provided complementary information on a biochemical properties of a cartilage repair tissue [17]. The dGEMRIC index, i.e., the T1 relaxation time following (Gd-DTPA)^2-^ administration (T1(Gd)), is an indirect measure of the glycosaminoglycan (GAG) concentration of cartilage tissue [18], [19], [20].

At field-strengths of 3 T, the biochemical MRI measurement of smaller joint cartilage, such as the ankle joint or lumbar facets, becomes possible in satisfactory image resolution and clinically reasonable measurement time [21], [22], [23]. Recently, these biochemical techniques were adapted to fibrocartilaginous tissues, such as the menisci [24], [25], where, similar to the fibrocartilage structure of the TMJ disc, GAGs are less abundant compared to hyaline cartilage [2], [26]. Recent results showed that T2 mapping technique enables ultrastructural analysis of the composition of the TMJ disc and is feasible in vivo [24].

Developed for hyaline cartilage, dGEMRIC imaging is an important step towards noninvasive compositional cartilage imaging, because it can show the biochemical ultrastructure of healthy and diseased cartilage. Different studies have demonstrated the ability of dGEMRIC to detect changes in cartilage degeneration before morphological changes occur, in early-stage osteoarthritis (OA) [27], [28]. The dGEMRIC method can also be used for the monitoring of the maturation of repair tissue after different cartilage repair surgeries [25], [29] and for longitudinal cohort evaluation of cartilage regeneration [30].

To our best knowledge, no dGEMRIC feasibility studies have been done yet on the disc of the TMJ.

The aim of this study was threefold: To 1) test the feasibility of delayed Gadolinium-enhanced Magnetic Resonance Imaging of Cartilage (dGEMRIC) at 3 T in the TMJ and 2) to determine the optimal delay for measurements of the TMJ disc after i.v. contrast agent (CA) administration.

## 2 Method

### 2.1 *In vivo* MRI

#### 2.1.1 Volunteers and study design

**Fig. 1:**
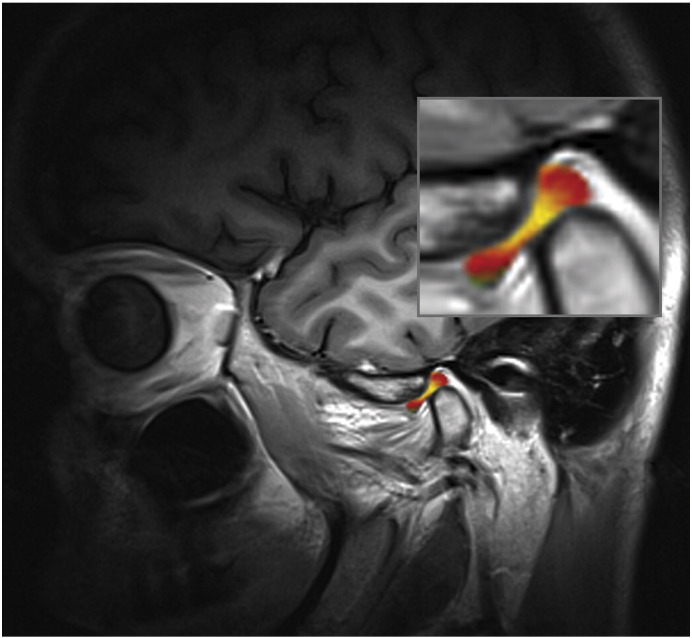
Overlay of T1-weighted image with pseudo-colored IR T1 maps of the TMJ in an asymptomatic volunteer. The TMJ is zoomed for better delineation of the anterior, central, and posterior parts of the TMJ. The central part of the TMJ demonstrates a lower GAG content.

**Fig. 2:**
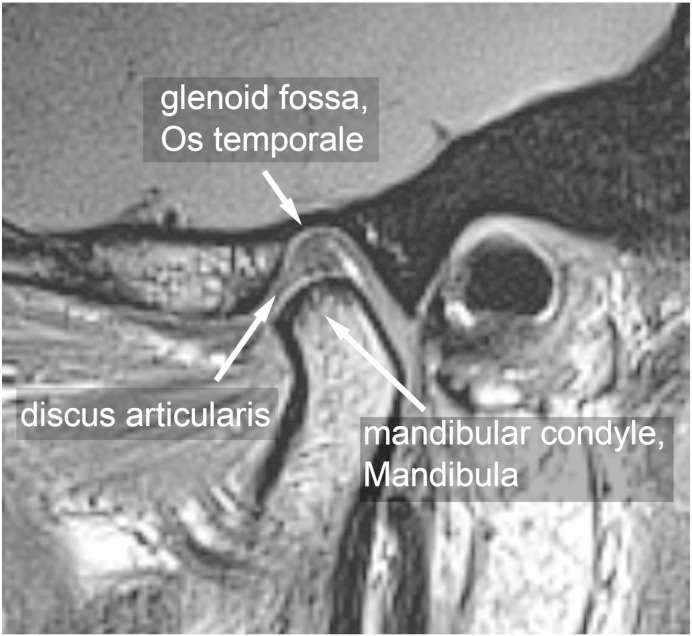
Image of the TMJ shows the morphology of the TMJ in an asymptomatic volunteer.

**Fig. 3:**
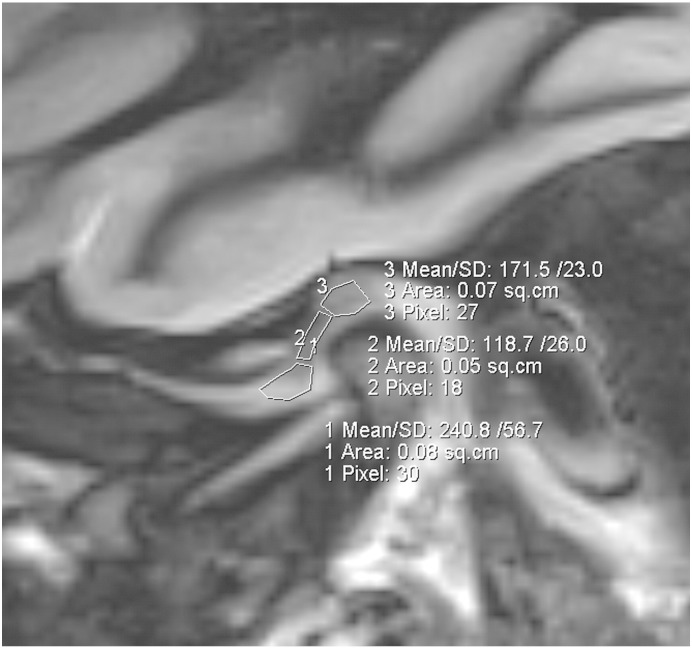
Example of three ROI evaluations of an image obtained using an IR sequence. Anterior, central, and posterior regions of interest were manually drawn and subsequently transferred from one time-stamped post contrast image to another using the “copy&paste” function. ROI values were fitted offline, using Interactive Data Language (IDL) software and T1(GD) values were summarized.

**Fig. 4:**
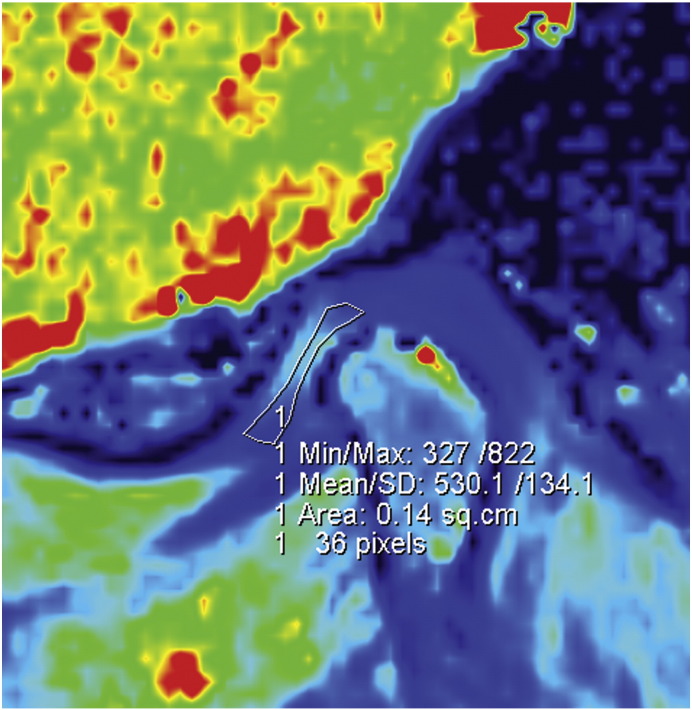
Example of an ROI on an image measured by the 3D GRE dual flip angle T1 technique. The image was pseudo-colored to increase contrast between individual TMJ regions.

Six volunteers were included in the study (two men, four women; age range, 20.8–28.1 years; mean BMI of 21.95, BMI range, 20.03–24.22). Volunteers were recruited both at the Bernhard-Gottlieb University Clinic of Dentistry, Department of Orthodontics and at the High Field MR Centre of the Medical University of Vienna. The local ethics committee approved this study and all volunteers gave written, informed consent. Prior the inclusion of the volunteers into the study, TMJ status of each individual volunteer was inspected by the experienced radiologist (S.T. – 18 years of experience in radiology). Only those volunteers, which were clinically asymptomatic and had physiological disc position, were enrolled into this study. MR examinations were performed on a 3 T whole-body Magnetom TimTrio scanner (Siemens Healthcare, Erlangen, Germany) equipped with gradient coils that provided a gradient field of 40 mT/m, slew rate of 200 mT/m/s. Volunteers lay supine with the head fixed to the flexible eight-channel multi-element coil (Noras, Würzburg, Germany). Coil elements were in close touch with the volunteer`s face, preventing motion of the volunteer`s head during the exam. A bolus of a double dose 0.2 mmol/kg of Gd-diethylenetriamine pentaacetic acid ion (Gd-DTPA)^2-^, i.e. 0.4 mL of Magnevist™ per kg body weight (Bayer Vital GmbH, Leverkusen, Germany) was administered to the volunteers after the initial native measurement. A parasagittal slice orientation was used in the inversion recovery as well as the 3D-GRE technique (Fig. 1). Fig. 2 shows the morphology of the TMJ. Three volunteers were examined using 2D inversion recovery protocols (Fig. 3), and the other three volunteers were examined using a 3D-GRE dual flip angle technique (3D-GRE). Fig. 4 shows an example of a T1 map calculated from the data measured by the 3D-GRE dual flip angle technique.

#### 2.1.2 Magnetic resonance imaging

For the 2D inversion recovery sequences, the MR protocol consisted of nine different inversion recovery measurements, with a 2D multi-slice, inversion recovery, spin-echo technique with inversion times as follows: [60, 100, 200, 300, 400, 500, 1000, 1500, 2500 ms]; TR set to 5000 ms; TE of 8.1 ms; number of slices 4; slice thickness 3 mm; spectral width 260 Hz/pixel; matrix size 384 × 384; flip angle 180 degrees; pixel resolution 0.52 × 0.52 mm; a total acquisition time of 2 min 57 s for IR of 60 ms, up to 4 min 23 s for an IR time of 2500 ms; and an FOV of 199 × 199 mm. The TR parameter increased with inversion time increases, maintaining TR constant.

In order to perform rapid (fine time-resolved) contrast agent uptake measurement, fast 3D-GRE was performed. The Siemens built-in B1 mapping was automatically performed before the first 3D-GRE measurement. The resulting B1 map was used for automatic image correction. This is particularly important for the 3D dual flip angle gradient echo technique, which is known to be sensitive to B1 inhomogeneities [25]. Measurement parameters were as follows: TR was set to 8 ms; TE was 2.5 ms; number of averages was 4; slice thickness 2 mm; spacing between slices was 0.4 mm; matrix size 320 × 320 pixels; low flip angle excitation pulse was automatically set to 4 degrees; the high flip angle excitation pulse was automatically set to 21 degrees (setting of excitation pulse flip angles was based on a prior estimate of expected T1 values); pixel resolution was 0.625 × 0.625 mm; total measurement time was 2 min 34 sec × 2; FOV was 200 × 200 mm; pixel bandwidth was 161 Hz/pix; and number of slices was 16.

#### 2.1.3 Image analysis/data evaluation

For the 2D inversion recovery sequences, one region of interest (ROI) was drawn on the shortest inversion time image, covering the entire TMJ disc, and subsequently 3 ROIs were drawn separately on the anterior, middle and posterior parts of the TMJ disc, using the Syngo Siemens built-in standard evaluation software. The ROIs were copied and pasted onto the rest of the inversion time images. Signal intensities in each ROI were recorded and T1 maps were calculated offline, using an IDL fitting routine based on the curvefit IDL code (by Craig B. Markwardt, NASA/GSFC Code 662, Greenbelt, MD 20770, craigm@lheamail.gsfc.nasa.gov).

For the 3D-GRE dual flip angle technique, T1 maps were calculated online using the built-in Syngo Siemens software. All cases were analyzed, and ROIs were drawn by one observer (E.P., a dentist who has specialized in orthodontics for four years and TMDs for three years). ROIs were manually defined on the disc of the right and left TMJ, and three ROIs (anterior, central, posterior) each were manually defined for different parts of the disc (Fig. 3). The observer attempted to include as many pixels as possible into the ROIs, to diminish non-systematic errors. The range of ROI sizes was between 0.25 cm^2^ (92 pixels) and 0.13 cm^2^ (48 pixels).

In order to compare T1 values measured by IR (30 and 60 minute intervals) with 3D GRE (8÷10 minute intervals), interpolation of the 3D GRE data was performed. The 3D GRE data measured in 8–10 minute intervals were interpolated into one minute intervals. Subsequently to match the IR time points (30 and 60 minute intervals), the GRE time points at 30 or 60 minutes were selected from interpolated data.

Interpolation was performed using IDL software (RSI, Boulder, CO), using built-in SPL_INTERP” routine, which provides spline interpolation over the measured dataset in selected time points. SPL_INTERP is based on the routine “spline” described in section 3.3 of *Numerical Recipes in C: The Art of Scientific Computing* (Second Edition), published by Cambridge University Press, and is used in IDL by permission.

#### 2.1.4 Statistical analysis

All statistical evaluations were performed using IBM SPSS Statistics Version 19.0. Metric data, such as T1 values, are presented using mean +/− SD. Mean values and standard deviation for each ROI were recorded and statistically analyzed using a two-way ANOVA for repeated measures. The two intersubject factors were “time point” and “side.” Post hoc pair-wise comparisons were performed using a Bonferroni correction. A p value equal to or below *p* = 0.05 was considered to indicate significant results.

## 3 Results

**Fig. 5:**
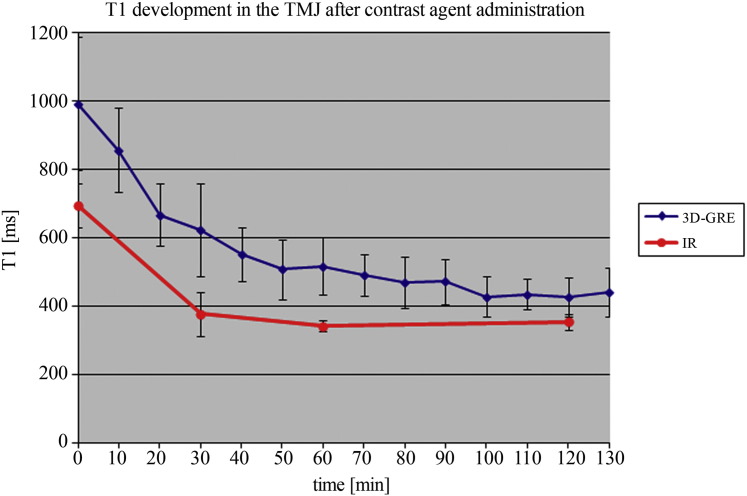
Mean T1 values of T1 (IR and 3D GRE) after contrast agent application. After 30 minutes, a statistically significant drop in T1 values was observed.

**Fig. 6:**
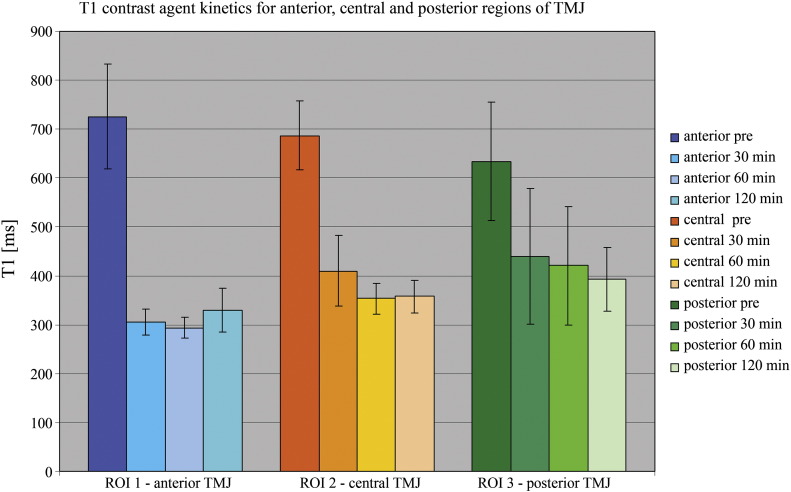
Summary bar graph of the contrast agent kinetics measured by inversion recovery. T1 for the anterior (ROI1), central (ROI2), and posterior (ROI3) regions of the TMJ disc (error bars show standard deviations).

The 2D inversion recovery sequences show a statistically significant drop (*p* < 0.001) in T1 from pre-contrast (T1_0_ = 688.5 ms) to 30 minutes post-contrast (*p* < 0.001; T1_30_ = 396.9 ms), and to 60 minutes post-contrast (*p* < 0.001; T1_60_ = 341.4 ms), as well as from T1 pre-contrast to 120 minutes post-contrast (*p* < 0.001; T1_120_ = 351.9 ms). A T1 drop of 50% was reached at time point 2, which was 60 minutes after contrast agent administration (Fig. 5, Fig. 6).

The 3D gradient echo sequences confirmed these results, with a significant drop in T1 between time point 0 and time point 1 (p < 0.001, T1_0_ = 992.1 ms, T1_10_ = 855.9 ms), and reaching a T1 drop of 50% between time points 6 (T1_60_ = 516.9 ms) and 7 (T1_70_ = 489.7 ms), after contrast agent administration (Table 1, Fig. 6).

**Table 1:** Estimated marginal means recalculated according to the 10-minute time intervals of all 13 time points measured by one observer.

Measur. num	base	1	2	3	4	5	6	7	8	9	10	11	12	13
Timepoint [min]	0	10	20	30	40	50	60	70	80	90	100	110	120	130
Mean T1 [ms]	992.1	855.9	666.1	623.8	5510.0	506.5	516.9	489.7	468.7	470.9	426.9	434.3	425.4	439.9
S.D.	195.3	123.8	91.7	136.6	78.9	87.8	82.9	60.6	74.5	65.4	58.2	45.5	58.3	72.5

When the 2D inversion recovery sequences were analyzed for differences within the TMJ disc, interestingly, all six TMJ discs showed the lowest T1 values in the anterior portion of the disc. In the central and posterior part of the disc, the results were heterogeneous. The tendency toward higher T1 values for the left TMJ can be explained by measurement time points – the left TMJ was measured first by default.

Despite known risk for NSF, as a side effect of dGEMRIC, we did not observe any complications after intra-venous contrast agent administration.

## 4 Discussion

To our knowledge, no attempt has been made to test the feasibility of dGEMRIC for GAG-specific biochemical MR imaging in the fibrocartilaginous disc of the TMJ to date. One recent case study of two volunteers and one cadaver focused on T2* values of the TMJ disc [26].

Recently, quantitative evaluation of the T1 relaxation times of the menisci following (Gd-DTPA)^2-^ administration was used to assess the potential of this technique for the detection of degenerative changes in fibrocartilage [31]. Long-term contrast agent kinetics of (Gd-DTPA)^2-^ in the menisci were measured in another study in asymptomatic volunteers for nine hours, with a suggested suitable time-window between 2.5 and 4.5 hours after contrast agent administration [32]. In our study, the optimal time window after i.v. contrast agent administration was between 60 and 120 minutes, which may be due to the different anatomical conditions (upper and lower joint space for contrast agent penetration compared to hyaline cartilage with only one surface to the joint space) and the more sensitive region of the TMJ area.

T1 reference values from knee cartilage (T1(Gd) = 636.0 ± 181.0 ms) [33] and from meniscal tissue (T1(Gd), 90 minutes after contrast agent administration = 660.0 ± 93. 8 ms) [34], are higher compared to our results in the fibrocartilaginous TMJ disc (T1(Gd) = 341.4 ms with 2D inversion recovery and 471.2 ms with 3D-GRE 60 minutes after contrast agent administration).

The mean signal intensity drop of 50% was reached 60 minutes after bolus injection in the TMJ disc, compared to a nearly 40% drop in meniscal tissue intensity after three hours [32]. Contrast agent kinetics in the TMJ disc seem to be substantially different compared to the fibrocartilage of the menisci.

The limitations of this feasibility study are the low number of asymptomatic volunteers included. In order to specify the drop in T1 values more precisely and also recommend time frame for post-contrast agent T1 measurement more precisely, the number of subjects should be higher in future studies. The aim of this study was to test the feasibility of measuring contrast agent kinetics in asymptomatic volunteers to provide a clinical time frame for the best dGEMRIC measurements of the TMJ disc in patients. In contrast to other studies on contrast agent kinetics in cartilage, the volunteers were not instructed to move the mandible for a faster uptake of contrast agent.

The use of double dose (0.2 mmol/kg) Gadolinium-based contrast agent pose another limitation of our study. According to the updated ESUR Contrast Medium Safety Committee guidelines [35] single dose (0.1 mmol/kg) Gadolinium-based contrast agent should be used. The ESUR Contrast Medium Safety Committee guidelines pose a regularly updated evidence for reducing the risk of Nephrogenic systemic fibrosis (NSF), which is associated with the intravenous application of a gadolinium based contrast media during dGEMRIC. The potential long-term problems from retention of small amounts of free gadolinium in the body after procedures enhanced with gadolinium-based contrast media are also considered [35].

In addition, these preliminary results with the three ROI evaluations within the TMJ disc provided an initial regional analysis of the contrast agent distribution within the disc, and thus, differences in the GAG content in different regions of the normal articular disc. The individual variations, even at time point T1_30_, could be due to individually different functional loading of the TMJ.

Biochemical MR may lead to a better understanding of the important biomechanical role of the TMJ, its different pathologies and could, in the long term, be useful in monitoring of the patients after different therapeutic procedures for different TMDs.

The preliminary results of our study showed that T1(Gd) maps calculated from 2D inversion recovery and 3D-GRE sequences are feasible for the *in vivo* assessment of the fibrocartilage disc of the TMJ. Similar to articular cartilage, but unlike preliminary results from the meniscal tissue, there seems to be a plateau for contrast agent uptake, starting 60 minutes after administration. The beginning of this plateau may be considered a suitable time point for dGEMRIC-like T1 mapping of the TMJ disc, even though the 3D gradient echo sequences indicate a statistically significant T1 drop earlier. Larger studies are required to investigate the differences in Gd-DTPA^2-^ kinetics in healthy and degenerative TMJ discs. The results of this study could provide the basis for further patient studies that focus on imaging of early degeneration and monitoring of different therapy measures.

The time required for IR is long and represents a clinical and practical limitation. On the other hand, 3D GRE technique is much faster, therefore more suitable for clinical application, although sensitivity to the B1 inhomogeneities has to be considered. The future application of the dGEMRIC to ultra-high field MR systems (7 T) could provide higher nominal image resolution in a given measurement time. This could further increase precision of the evaluation of small structures like cartilage of a TMJ disc. However, 7 T systems are currently exclusively experimental devices.

In conclusion, our study show 1) the feasibility of dGEMRIC at 3 T in the TMJ and 2) the optimal delay for the measurements of the TMJ disc after iv CA administration is 60 minutes.
